# Cytoplasmic, nuclear, and total PBK/TOPK expression is associated with prognosis in colorectal cancer patients: A retrospective analysis based on immunohistochemistry stain of tissue microarrays

**DOI:** 10.1371/journal.pone.0204866

**Published:** 2018-10-04

**Authors:** Tzu-Cheng Su, Chun-Yu Chen, Wen-Che Tsai, Hui-Ting Hsu, Hsu-Heng Yen, Wen-Wei Sung, Chih-Jung Chen

**Affiliations:** 1 Department of Surgical Pathology, Changhua Christian Hospital, Changhua, Taiwan; 2 Department of Surgery, National Taiwan University Hospital, Taipei, Taiwan; 3 Department of Otorhinolaryngology, Head and Neck Surgery, Changhua Christian Hospital, Changhua, Taiwan; 4 School of Medicine, Chung Shan Medical University, Taichung, Taiwan; 5 Institute of Medicine, Chung Shan Medical University, Taichung, Taiwan; 6 Department of Gastroenterology, Changhua Christian Hospital, Changhua, Taiwan; 7 Department of Urology, Chung Shan Medical University Hospital, Taichung, Taiwan; 8 Department of Medical Technology, Jen-Teh Junior College of Medicine, Nursing and Management, Miaoli, Taiwan; Institut national de la recherche scientifique, CANADA

## Abstract

**Objective:**

PDZ-binding kinase/T-LAK cell-originated protein kinase (PBK/TOPK) regulates components of the cell cycle, including cell growth, immune responses, DNA damage repair, apoptosis, and inflammation. PBK/TOPK may also accelerate tumorigenesis in colorectal cancer.

**Methods:**

We investigated the impact of PBK/TOPK on the clinical outcome of colorectal cancer patients to further identify its role in colorectal cancer. PBK/TOPK immunoreactivity was analyzed by immunohistochemistry in 162 cancer specimens from primary colorectal cancer patients.

**Results:**

The mean follow-up time after surgery was 5.4 years (medium: 3.9 years; range 0.01 to 13.1 years). The prognostic value of PBK/TOPK on overall survival was determined by Kaplan-Meier analysis and Cox proportional hazard models. PBK/TOPK was expressed in both the cytoplasm and nucleus. High PBK/TOPK expression in tumor cells was significantly associated with advanced T value. The 5-year survival rate was greater for patients with high total PBK/TOPK expression than with low PBK/TOPK expression (58.3% vs 34.4%, P = 0.005). Multivariate analyses showed that low-scoring cytoplasmic PBK/TOPK, negative nuclear PBK/TOPK, low total PBK/TOPK, and advanced tumor stage were correlated with poor overall patient survival.

**Conclusions:**

We suggest that PBK/TOPK expression, detected by IHC staining, could be used as an independent prognostic marker for colorectal cancer patients.

## Background

Colorectal cancer (CRC) is the third major cause of cancer mortality and morbidity worldwide. Despite rising incidence of CRC, the overall mortality tends to be decreased in recent years, likely due to the early detection and improved treatment strategy [1, 2]. The oncogenesis of CRC is positively correlated with age, alcoholism, smoking, excessive red meat and fat consumption, family history of CRC, and the presence of genes with potential to precipitate chronic intestinal diseases [3].

Several prognostic biomarkers and genes have been reported to have some benefit to CRC patients by permitting early detection and ensuing treatment, which can improve their prognosis [4–7]. However, clinicians still encounter difficulties when attempting to choose a definitive approach for curing CRC patients or significantly improving their prognosis, largely due to the diverse oncogenesis that leads to cancer progression and metastasis [7, 8]. Much research is now attempting to unveil references that can reveal the degree of a particular malignancy. Thus, a great demand exists for novel noninvasive biomarkers that can aid in the diagnosis of CRC malignancies with high recurrence rates or metastatic potential [7, 8].

PDZ-binding kinase/T-LAK cell-originated protein kinase (PBK/TOPK) is a 322 amino acid mitogen-activated protein kinase (MAPK) kinase (MAPKK)-like serine/threonine kinase that regulates cell cycle processes, including cell growth, immune responses, DNA damage repair, apoptosis, and inflammation [9–12]. This protein is barely detectable in normal somatic tissues, except for testicular and embryonic tissues and proliferating brain neural stem cells. Many studies have shown overexpression of PBK/TOPK in malignancies such as Ewing sarcoma, lymphoma, leukemia, melanoma, breast cancer, lung cancer, cholangiocarcinoma, and CRC [13–16].

Previous research has shown that the complex of PBK/TOPK and cdk1/cyclin B1 promotes cytokinesis by phosphorylation of protein regulator cytokinesis 1 (PRC1) [17]. The subsequent downstream targets of PBK/TOPK include two MAPKs (JNK1 and ERK2); ERK2 forms a positive feedback loop with PBK/TOPK, which may facilitate the initiation of CRC during mitotic phase of the cell cycle by enhancing the polymerization of microtubules [12]. PBK/TOPK also may accelerate tumorigenesis in CRC through inhibition of p53 expression [10]. Although the expression of PBK/TOPK is closely related to the malignancies mentioned above, its prognostic role still remains complicated and unclear.

Previous studies have rarely confirmed a correlation between PBK/TOPK expression and the clinicopathological features of CRC. In this study, we provide a correlation between PBK/TOPK expression and clinicopathological features and show that this correlation has prognostic significance for CRC patients, as well as providing a capability to assess their clinical outcome.

## Materials and methods

### Study subjects and ethics statement

Tumor tissues were collected from 162 patients with confirmed histological CRC diagnosis at Changhua Christian Hospital between 1997 and 2000. Patients with history of other malignancy or missing clinical data were excluded. Among 162 patients, 136 died during the follow up survey. Cancers were staged according to the AJCC Colon Cancer Staging, 7th edition (2010). Clinical data, including gender, age, stage, T, N, and M stages, and follow-up information, were obtained from medical records and the cancer registry. This study was approved and the consent was waived by the Institutional Review Board and the Ethics Committee of the Changhua Christian Hospital, Changhua, Taiwan (IRB no. 121008).

### Immunohistochemistry staining and evaluation of PBK/TOPK immunoreactivity

Immunohistochemistry (IHC) staining was performed at the Department of Surgical Pathology, Changhua Christian Hospital, as previously described [18, 19]. IHC analyses were performed on tissue microarray sections (4 μm) of formalin-fixed, paraffin-embedded, pre-chemotherapy primary colorectal tumors. The antibody used was anti-human PBK/TOPK (PBK/TOPK antibody, sc-136026, 1:150 dilution, Santa Cruz Biotechnology). Immunoreactivity was analyzed by pathologists, using a previously described scoring system [18, 20]. Briefly, immunoreactivity scores were defined as the cell staining intensity (0 = nil; 1 = weak; 2 = moderate; and 3 = strong) multiplied by the percentage of stained cells (0–100%), leading to scores from 0 to 300. Positive and negative control staining were shown in S1 Fig.

### Statistical analysis

The Student *t* test, Fisher’s exact test, and the χ^2^ test were applied for continuous or discrete data analysis. The associations between PBK/TOPK score and patient survival were estimated using the Kaplan–Meier method and assessed using the log-rank test. Potential confounders were adjusted by Cox regression models, with the PBK/TOPK score fitted as indicator variables. Overall survival time was defined as the interval between the date of surgery and the date of last follow-up or death. All statistical analyses were conducted using the SPSS statistical software program (version 15.0) (SPSS, Inc., Chicago, IL). All statistical tests were 2-sided, and the values of *P* <0.050 were considered statistically significant.

## Results

### Patient characteristics

Overall, 162 patients (92 males and 70 females) with a mean age of 64.3 ± 13.2 years (range from 22–93 years) were enrolled in this retrospective study. The histological tumor type of all 162 patients was adenocarcinoma with 152 patients of non-mucinous and 10 patients with mucinous type. As to the tumor location, 56 patients had tumor located in the rectum, 26 patients with tumor located in the sigmoid. Other locations included 14 in the ascending, 10 in the rectosigmoid, 8 in the descending, 4 in the cecum, and 1 in the transverse (43 missing data). In total, 133 patients had early stage tumors (I+II+III) and 29 patients had advanced stage tumors (IV). Thirty-one (19.1%) patients had tumor metastasis (M1). The overall survival time ranged from 0.01 to 13.1 years, with a mean survival time of 5.1 years.

### Correlation between PBK/TOPK expression and clinicopathological features

**Fig 1:**
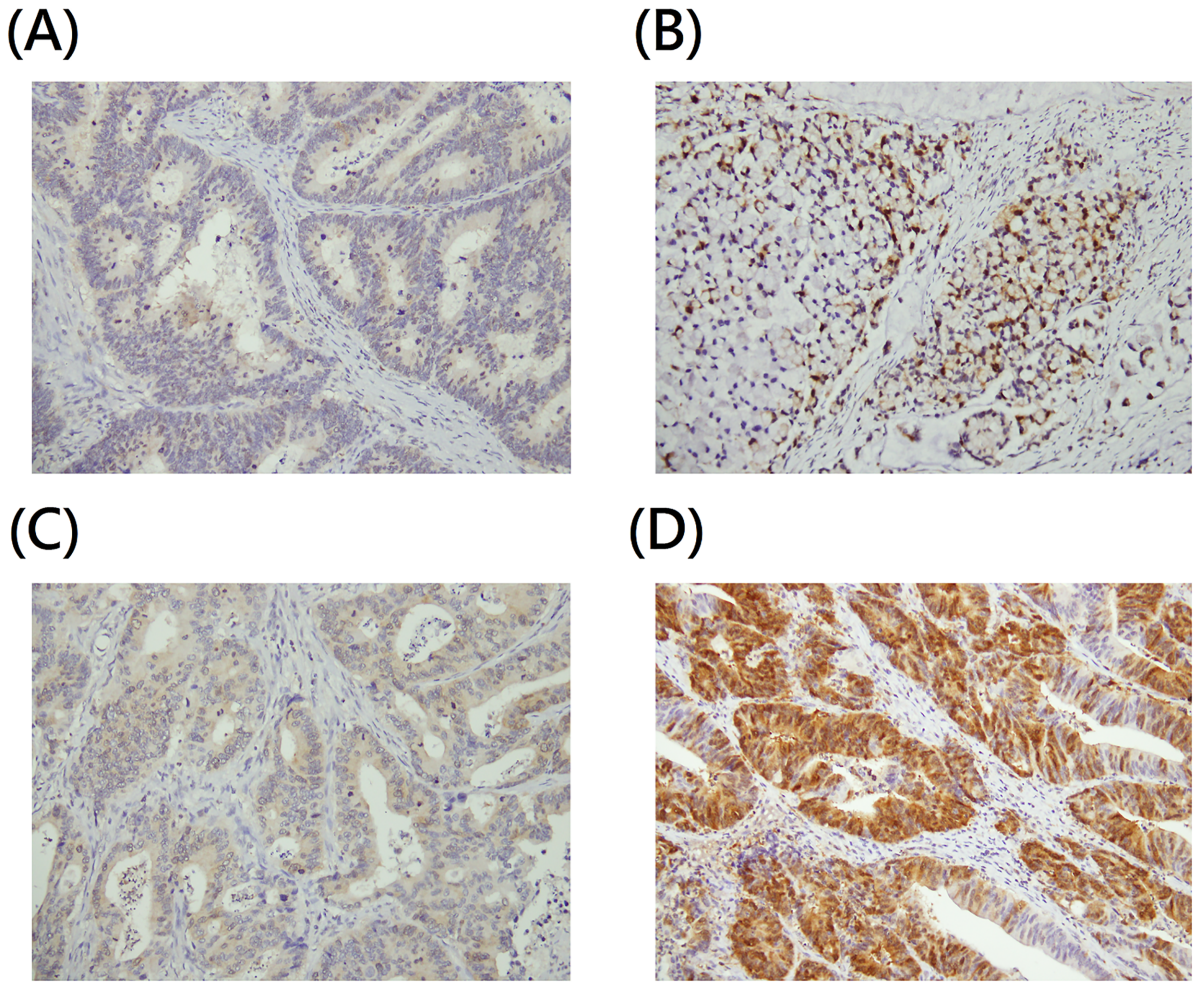
Representative immunostaining of PBK/TOPK in tissue arrays of colorectal cancer specimens. PBK/TOPK expression levels (cytoplasmic/nuclear) were (A) low/negative; (B) low/positive; (C) high/negative; (D) high/positive.

PBK/TOPK was expressed in the cytoplasm and nucleus. Representative images of IHC staining are shown in Fig 1. The cytoplasmic PBK/TOPK expression score was 74.3±61.3 (mean±SD) and the median value was 70. Therefore, we defined cytoplasmic PBK/TOPK expression level <70 as low expressions. We analyzed nuclear PBK/TOPK expression in negative (57 cases) and positive (105 cases) groups. The expression score for nuclear PBK/TOPK was 23.7±24.2 (mean±SD) with median value of 10. We combined the expression level of PBK/TOPK in the cytoplasm and nucleus into a total PBK/TOPK expression score. Patients with high cytoplasmic PBK/TOPK and positive nuclear PBK/TOPK were defined as having high total PBK/TOPK expression.

The relationships between various clinical parameters and PBK/TOPK expression are listed in Table 1. Among all parameters, high cytoplasmic expression and high total PBK/TOPK expression in tumor cells were significantly associated with a high T value (P = 0.015). In particular, a significant association was noted between a high T value and high total PBK/TOPK expression (P = 0.019). However, no significant association was noted between PBK/TOPK expression and age, gender, lymph node metastasis, distant metastasis, or tumor stage.

**Table 1:** Relationships of PBK/TOPK expression with clinical parameters in 162 colorectal cancer patients.

Parameters	Case number	Cytoplasm PBK/TOPK		p value	Nucleus PBK/TOPK		p value	Total PBK/TOPK		p value
		Low	High		Negative	Positive		Low	High	
Age (year)		62.5±12.6	66.0±13.6	0.095	63.6±13.6	64.6±13.0	0.631	63.3±13.1	65.4±13.4	0.320
Gender										
Female	70	28 (40.0)	42 (60.0)	0.052	24 (34.3)	46 (65.7)	0.834	35 (50.0)	35 (50.0)	0.215
Male	92	51 (55.4)	41 (44.6)		33 (35.9)	59 (64.1)		55 (59.8)	37 (40.2)	
LN involvement										
No	82	36 (43.9)	46 (56.1)	0.210	24 (29.3)	58 (70.7)	0.110	40 (48.8)	42 (51.2)	0.079
Yes	80	43 (53.8)	37 (46.2)		33 (41.3)	47 (58.8)		50 (62.5)	30 (37.5)	
Stage										
I+II+III	133	63 (47.4)	70 (52.6)	0.446	43 (32.3)	90 (67.7)	0.103	72 (54.1)	61 (45.9)	0.436
IV	29	16 (55.2)	13 (44.8)		14 (48.3)	15 (51.7)		18 (62.1)	11 (37.9)	
T value										
1+2	26	7 (26.9)	19 (73.1)	0.015	5 (19.2)	21 (80.8)	0.063	9 (34.6)	17 (65.4)	0.019
3+4	136	72 (52.9)	64 (47.1)		52 (38.2)	84 (61.8)		81 (59.6)	55 (40.4)	
N value										
0	95	45 (47.4)	50 (52.6)	0.672	30 (31.6)	65 (68.4)	0.252	50 (52.6)	45 (47.4)	0.372
1+2	67	34 (50.7)	33 (49.3)		27 (40.3)	40 (59.7)		40 (59.7)	27 (40.3)	
M value										
0	131	61 (46.6)	70 (53.4)	0.249	42 (32.1)	89 (67.9)	0.087	70 (53.4)	61 (46.6)	0.264
1	31	18 (58.1)	13 (41.9)		15 (48.4)	16 (51.6)		20 (64.5)	11 (35.5)	

### Prognostic value of PBK/TOPK expression in CRC tumor tissues

**Fig 2:**
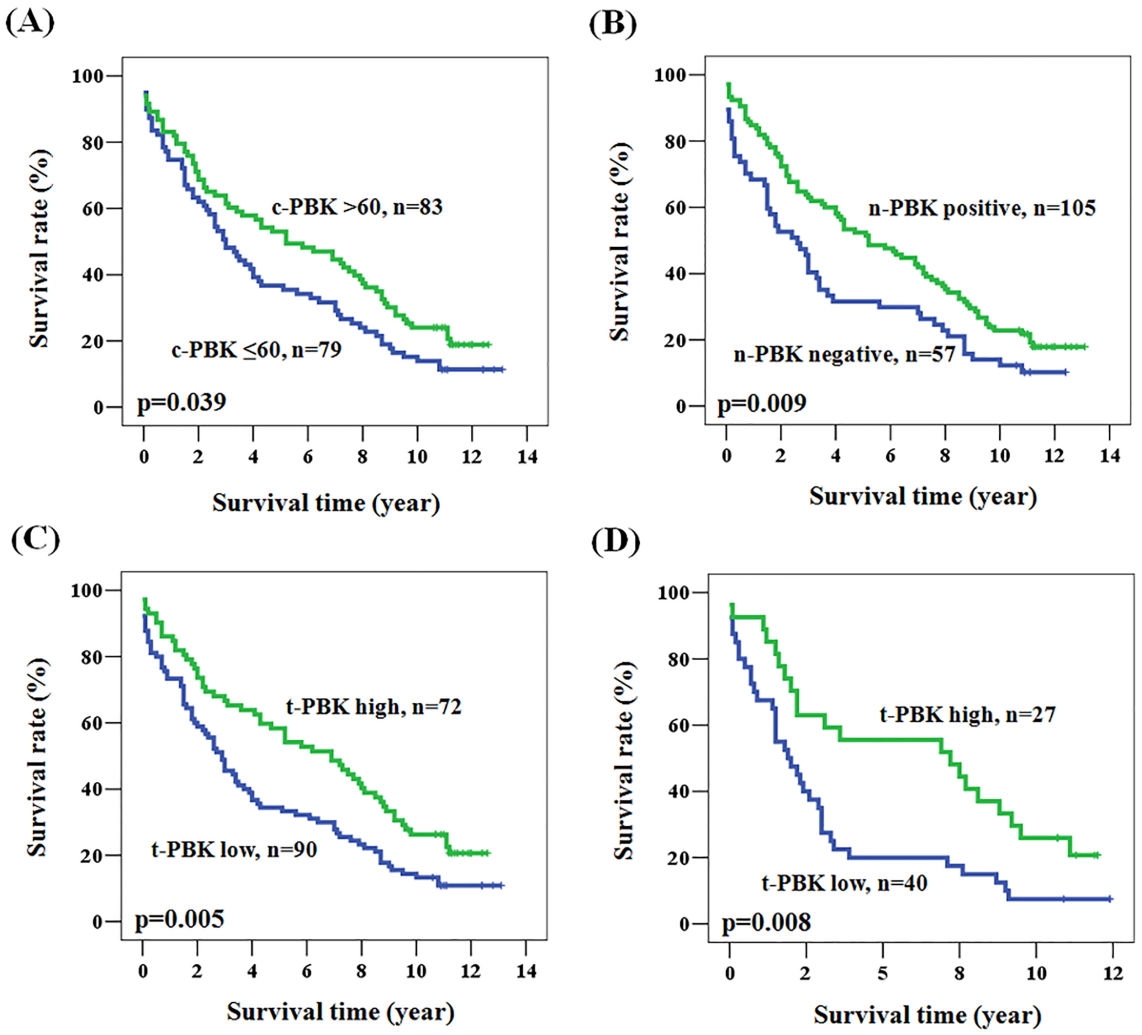
Kaplan-Meier actuarial analysis of PBK/TOPK expression with respect to overall survival of patients according to expression of (A) c-PBK/TOPK, (B) n-PBK/TOPK, (C) t-PBK/TOPK, and (D) t-PBK/TOPK of patients with N = 1+2.

The Kaplan-Meier survival curves demonstrated a relationship between patient prognosis and PBK/TOPK expression (Fig 1). We evaluated the prognostic impacts of various parameters by univariate and multivariate analyses (Tables 2 and 3). The univariate analysis revealed that advanced disease stage, low cytoplasm PBK/TOPK expression, negative nuclear PBK/TOPK expression, and low total PBK/TOPK expression were significantly associated with poor overall patient survival (P = 0.023, P = 0.042, P = 0.011, and P = 0.005, respectively, Table 2). As expected, patients with advanced stage disease had poorer 5-year survival when compared with those with early stage disease (50.4% vs 20.7%, log rank P = 0.023). The 5-year survival rate was greater for patients with high PBK/TOPK expression than with low PBK/TOPK expression, based on cytoplasmic, nuclear, or total expression (5-year survival data are listed in Table 2 and Fig 2). Multivariate analysis was performed to identify whether PBK/TOPK expression could be an independent prognostic marker in our population. Low cytoplasmic PBK/TOPK expression (HR = 1.469, 95% CI: 1.038–2.081, P = 0.030), negative nuclear PBK/TOPK expression (HR = 1.604, 95% CI: 1.123–2.291, P = 0.009), and low total PBK/TOPK expression (HR = 1.737, 95% CI: 1.220–2.472, P = 0.002) were correlated with significantly poor overall patient survival, after adjusting for age, gender, and stage (Table 3). We also subdivided the patients according to various clinical parameters and used multivariate analysis to examine the influence of total PBK/TOPK expression (Table 4). The prognostic value of total PBK/TOPK expression was significant in patients with age≥65, positive lymph node involvement, advanced stage disease (including T and N value), and no distant metastasis. The advanced-staged patients had poorer prognosis if they also had low rather than high PBK/TOPK expression (HR = 2.332, 95% CI: 1.370–3.969, P = 0.002). The hazard ratio for overall survival in patients with lymph node involvement was 2.419 (95% CI = 1.345–4.348, P = 0.007) for low total PBK/TOPK expression compared to high total PBK/TOPK expression (Fig 2D). However, patients with distant metastasis showed no significantly different survival rate (P = 0.298) regardless of whether they had high or low total PBK/TOPK expression. These results showed that the prognostic value of PBK/TOPK was more significant in specific subgroups.

**Table 2:** Univariate analysis of the influence of various parameters on overall survival in colorectal cancer patients.

Parameter	Category	Overall survival			
		5-year survival (%)	HR	95% CI	p value
Age, y	≥65/<65	44.2/46.3	1.294	0.916–1.829	0.144
Gender	Male/Female	44.6/45.7	1.172	0.833–1.649	0.362
Stage	IV/I+II+III	20.7/50.4	1.658	1.071–2.568	0.023
Cytoplasm PBK/TOPK	Low/High	36.7/53.0	1.421	1.013–1.992	0.042
Nucleus PBK/TOPK	Negative/Positive	31.6/52.4	1.574	1.110–2.232	0.011
Total PBK/TOPK	Low/High	34.4/58.3	1.629	1.155–2.298	0.005

**Table 3:** Multivariate analysis of the influence of various parameters on overall survival in colorectal cancer patients.

Parameter	Category	Overall survival			
		5-year survival (%)	HR^1^	95% CI	p value
Age, y	≥65/<65	44.2/46.3	1.330	0.939–1.885	0.108
Gender	Male/Female	44.6/45.7	1.228	0.871–1.732	0.242
Stage	III+IV/I+II	33.8/55.3	1.685	1.087–2.613	0.020
Cytoplasm PBK/TOPK^2^	Low/High	36.7/53.0	1.469	1.038–2.081	0.030
Nucleus PBK/TOPK^3^	Negative/Positive	31.6/52.4	1.604	1.123–2.291	0.009
Total PBK/TOPK^4^	Low/High	34.4/58.3	1.737	1.220–2.472	0.002

^1^Adjusted for age, gender, and stage

^2^Adjusted stage: HR = 1.678, 95% CI = 1.082–2.601, p = 0.021

^3^Adjusted stage: HR = 1.593, 95% CI = 1.026–2.475, p = 0.038

^4^Adjusted stage: HR = 1.707, 95% CI = 1.101–2.648, p = 0.017

**Table 4:** Multivariate analysis of the influence of total PBK/TOPK expression according to clinical parameters on overall survival in colorectal cancer patients.

Parameter	Overall survival^1^			
	5-year survival (%)^2^	HR	95% CI	p value
All cases	34.4/58.3	1.798	1.260–2.566	0.001
Age (year)				
<65	37.2/62.5	1.383	0.783–2.442	0.264
≥65	31.9/56.3	2.094	1.335–3.285	0.001
Gender				
Female	37.1/54.3	1.963	1.128–3.418	0.017
Male	32.7/62.2	1.638	1.031–2.604	0.037
LN meta				
No	42.5/59.5	1.640	0.993–2.706	0.053
Yes	28.0/56.7	2.071	1.218–3.522	0.007
Stage				
I+II	47.8/64.1	1.433	0.882–2.329	0.146
III+IV	20.5/51.5	2.332	1.370–3.969	0.002
T value				
1+2	66.7/76.5	1.292	0.456–3.657	0.629
3+4	30.9/52.7	1.750	1.192–2.569	0.004
N value				
0	46.0/60.0	1.554	0.973–2.483	0.065
1+2	20.0/55.6	2.419	1.345–4.348	0.003
M value				
0	40.0/63.9	1.792	1.207–2.659	0.004
1	15.0/27.3	1.776	0.601–5.247	0.298

^1^Adjusted for age, gender, and stage

^2^Total PBK/TOPK expression: Low/High

## Discussion

CRC is one of the most common malignancies in the world. Most cases of CRC start as small, benign clumps called adenomatous polyps, with few other symptoms, making this cancer hard to detect in its beginning stages. However, current screening methods for CRC, including the test for occult blood and regular sigmoidoscopies (with consistent performance of polypectomy when needed), have increased the sensitivity and specificity of screening. The occult blood test can reduce the mortality of CRC by 14%, whereas the polypectomy technique can diminish the incidence of CRC by 50–70% [21, 22]. Nevertheless, the difficulty in diagnosis at early stages and the poor prognosis and complicated molecular mechanisms of CRC emphasize the urgency for developing a more thorough understanding of the biomarkers of CRC, in order to differentiate CRC patients with poor prognosis as early as possible and to increase the overall survival rate.

The 162 CRC patients recruited in the present study showed no significant differences between PBK/TOPK expressions and patient age, gender, lymph node metastasis, distant metastasis, or tumor stage. However, the patients with either advanced or positive lymph node CRC showed a clear relationship between low total PBK/TOPK expression and poor outcome. Overall, PBK/TOPK expression might be related to the cytogenesis of tumor cells, as we found that a lower expression of total PBK/TOPK gave a poorer prognosis for a CRC patient. Nevertheless, the complete role of PBK/TOPK in CRC still remains unclear.

We found that the PBK/TOPK appears to be overexpressed in different cellular locations (nucleus or cytoplasm) in different CRC patients. The CRC patients with low-scoring and cytoplasmic PBK/TOPK expression, with negative nuclear PBK/TOPK expression, with advanced stage tumors (stage IV), or with low total PBK/TOPK expression were revealed, for the first time, to have a relatively poor prognosis. These findings suggest that the benefit of using PBK/TOPK inhibitor might be limited due to the relatively favorable clinical outcome of patients with high PBK/TOPK expression. Even though PBK/TOPK inhibitor can significantly suppress tumor growth as well as increasing colon cancer cell apoptosis in cell model [23]. The underlying mechanism responses for our clinical finding is unclear and further analysis of under lying mechanism might solve this problem.

The studies of the correlations between PBK/TOPK and malignancies in recent years have confirmed that PBK/TOPK is overexpressed in proliferative cells. For example, PBK/TOPK is upregulated during the cytogenesis of tumor cells, most likely by phosphorylation of Thr9 and activation by cyclin B1/cdk1. Therefore, PBK/TOPK is hypothesized to play an important role in cytokinesis [17, 24, 25]. A recent study showed that PBK/TOPK down-regulation is significantly associated with poor prognosis in patients with cholangiocarcinoma [14]. Another study reported that PBK/TOPK expression was decreased due to EWS–FLI1 inhibition, which promoted a reduction in the cell proliferation rate and prevented the cells of Ewing sarcoma from growing and coalescing.

Parameters other than PBK/TOPK expression have also been found to affect tumorigenesis [3]. The present finding that low total PBK/TOPK expression is closely related to poor outcomes might be contrary to a previously hypothesized mechanism, which viewed PBK/TOPK as a gene related to cytogenesis that is overexpressed in tumor cells. Other signaling pathways probably exist that have not yet been identified and could affect the clinical course of CRC. Another explanation is that various parameters not considered in the present study are also closely connected with tumor growth. Otherwise, the role of PBK/TOKP might influence by the mutations or amplifications of oncogenes. Patients with high PBK/TOKP expression had unfavorable prognosis in sporadic patients with KRAS (KRAS proto-oncogene) or BRAF (B-Raf proto-oncogene) mutations [26]. However, there was no association between VEGFA (vascular endothelial growth factor A) amplification and KRAS gene status or with the PBK/TOKP protein expression [27]. These results suggest complex role of PBK/TOKP integration with gene mutation or amplification which might change the response of treatment targeting PB/TOKP K or the prognostic role of PBK.

Most cell cycle research has emphasized the timing of cell cycle regulator activation; however, the localization of the regulators also plays a pivotal role in the final activation. For instance, Cdc14, a nucleolar protein phosphatase present during most of the cell cycle, is able to cause the breakdown of its target, cyclin, only after release from the nucleolus during nuclear division [28, 29]. A key finding in the present study is that PBK/TOPK is present in both nuclear and cytoplasmic locations in CRC patients and that the location of PBK/TOPK has a significant influence on the clinical course in these patients. The CRC patients with low-scoring cytoplasmic PBK/TOPK expression and negative nuclear PBK/TOPK expression had the poorest prognosis. This is the first time that the location of PBK/TOPK expression in CRC patients has been linked to patient outcome. There are several limitations in this study. Considering the limited sample size and patients are from the same country, further investigations are necessary. Since this study is a retrospective analysis based on tissue microarrays, limited size of tissue microarray could not represent the protein expression of the whole tumor. Moreover, there is no information about the cancer specific death, adjuvant or neoadjuvant chemotherapy which would influence the prognosis. Otherwise, the surgical interventions were done between 1997 and 2000 which could not reflect the influence of the improved skills with advanced surgical devices or medications in the prognosis of CRC patients.

Previous studies have discussed the details of the mechanism and interactions of PBK/TOPK in the cell cycle [13, 15, 16, 24, 25]. The present study focused on the relationship between CRC cancer and PBK/TOPK expression, and especially on the localization of PBK/TOPK, and we concluded that expression and localization are correlated with the prognosis of CRC patients. Subsequent research should look for different mechanisms and functions of PBK/TOPK at different sites, with the aim of developing new screening methods or therapies for CRC.

## Conclusions

PBK/TOPK expression might be used as an independent prognostic marker for colorectal cancer patients. However, considering the limited sample size of this study, further investigation with larger population is necessary before clinical application as prognostic marker or therapeutic target.

## Supporting information

S1 Fig Representative PBK/TOPK IHC staining of (A) negative (colon tissue) and (B) positive (liver tissue) control. (DOCX)
