# Supplementary material for: Cytoplasmic, nuclear, and total PBK/TOPK expression is associated with prognosis in colorectal cancer patients: A retrospective analysis based on immunohistochemistry stain of tissue microarrays
Source: PLoS One. 2018 Oct 4;13(10):e0204866. doi: 10.1371/journal.pone.0204866 (PMC6171876; doi:10.1371/journal.pone.0204866)
Supplement: S1 Fig — (DOCX) [file pone.0204866.s001.docx]

Supporting Information


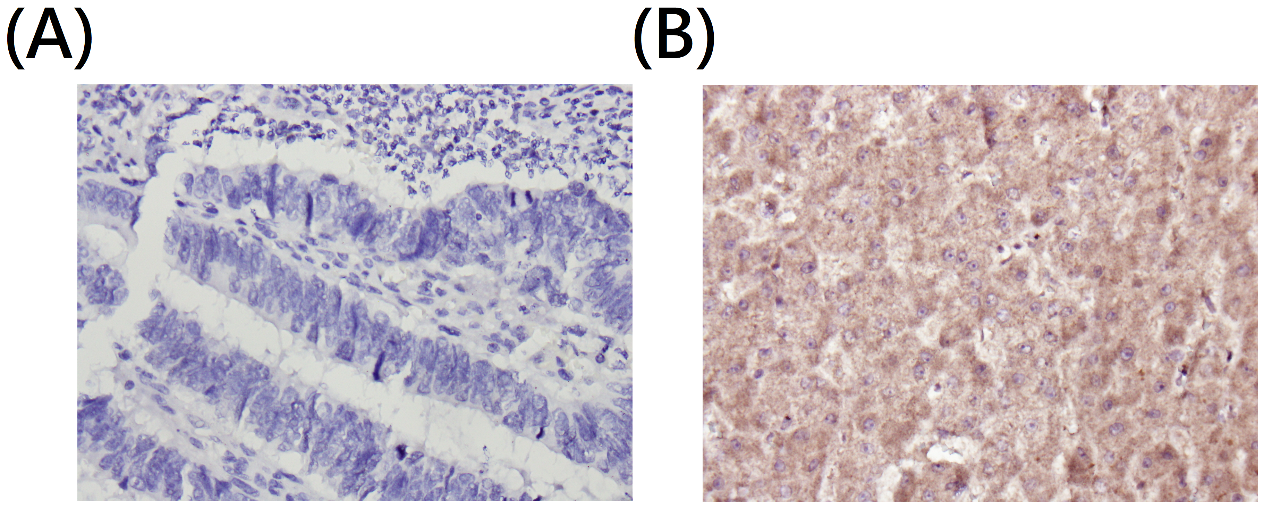


S1 Fig. Representative PBK/TOPK IHC staining of (A) negative (colon tissue) and (B) positive (liver tissue) control.
